# Current Understanding of IL-37 in Human Health and Disease

**DOI:** 10.3389/fimmu.2021.696605

**Published:** 2021-06-25

**Authors:** Zhangci Su, Xiaoan Tao

**Affiliations:** ^1^ Hospital of Stomatology, Sun Yat-sen University, Guangzhou, China; ^2^ Guanghua School of Stomatology, Sun Yat‐sen University, Guangzhou, China; ^3^ Guangdong Provincial Key Laboratory of Stomatology, Guangzhou, China

**Keywords:** IL-1 family, inflammation, regulatory cytokine, acquired immunity, cancer, autoimmune disease

## Abstract

IL-37 is a recently discovered cytokine in the IL-1 family exerting broad protective effects on inflammatory diseases, autoimmune diseases, and cancer. Immune and non-immune cells produce the IL-37 precursor upon pro-inflammatory stimuli. Intracellularly, caspase-1 cleaves and activates IL-37, and its mature form binds to Smad3; this complex translocates into the nucleus where it suppresses cytokine production, consequently reducing inflammation. Extracellularly, IL-37 forms a complex with IL-18Rα and IL-1R8 (formerly TIR8 or SIGIRR) that transduces anti-inflammatory signals by the suppression of NF-κB and MAPK and the activation of Mer-PTEN-DOK pathways. During inflammation, IL-37 suppresses the expression of several pro-inflammatory cytokine in favor to the expression of the anti-inflammatory ones by the regulation of macrophage polarization, lipid metabolism, inflammasome function, TSLP synthesis and miRNAs function. Moreover, IL-37 not only regulates the innate and acquired immunity, but also improves aging-associated immunosenescence. Furthermore, IL-37 exerts an inhibitory effect on tumor angiogenesis and metastasis, and progression. Finally, IL-37 may have a potential ability to reduce excessive inflammation since it is aberrantly expressed in patients with inflammatory diseases, autoimmune diseases, and cancer, thus, it may be used as a marker for different types of diseases. Therefore, this review provides an updated view of the role of IL-37 in human health and disease, and discusses the potential of IL-37 as a therapeutic target and biomarker in inflammatory diseases, autoimmune diseases, and cancer.

## Introduction

Human IL-37 is a newly discovered member of the IL-1 family has the ability to inhibit inflammation and immune response by inhibiting the production of pro-inflammatory cytokines, and ameliorate inflammation-induced fatigue by inducing metabolic reprogramming and limiting the metabolic effects of inflammation (1). IL-37 consists of five variants a, b, c, d and e, and it functions as an intracellular and extracellular cytokine. IL-37 is expressed and released in the cytosol in its pro-inactive form that requires cleavage to be transformed in its active form, and maturation and secretion are mediated by inflammatory caspases upon inflammasome signaling complexes (2). IL-37 was first identified in silico in 2000. The anti-inflammatory properties of IL-37 were first revealed by the group of Prof. Dinarello (3). Pro-inflammatory stimuli, including the ones triggered by cytokines, can induce the production of human IL-37, which is a self-protective mechanism against uncontrolled inflammation and excessive tissue damage. IL-37-deficient mice cannot be created to confirm its anti-inflammatory function since the IL-37 homologous gene has not been identified in the mouse, but it was confirmed by the generation of transgenic mice expressing the human IL-37 gene (IL-37-tg). Moreover, IL-37 not only regulates innate and acquired immunity, but also improves aging-associated immunosenescence. Furthermore, IL-37 can exert an inhibitory effect on cancer development and progression. Excellent reviews on this topic are available (4).

**Figure 1:**
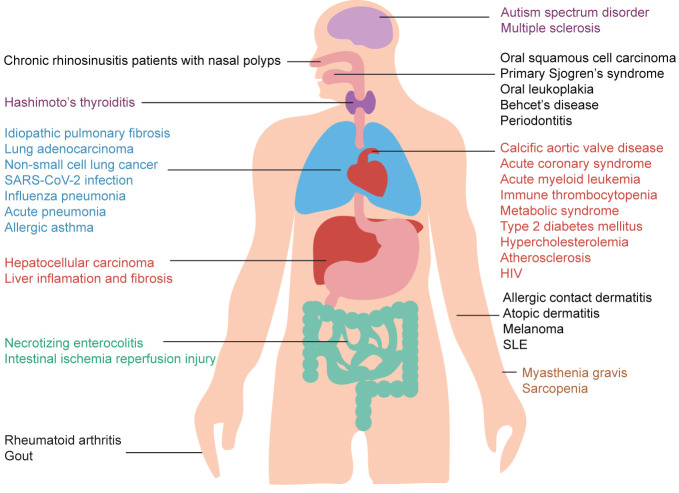
Potential roles of IL-37 in human health and disease. IL-37 exerts a wide range of protective effects in several different diseases. Moreover, IL-37 may be used as a biomarker for inflammatory diseases, autoimmune diseases, and cancer due to the abnormal levels of IL-37 in patients affected by these diseases.

Importantly, altered IL-37 expression in the serum was found in patients with different inflammatory diseases, autoimmune diseases, and cancer, as shown in **Figure 1**. The above discoveries increase the interest on the biological role of IL-37 also from a translational perspective, but despite that, certain biological properties and its precise role in human diseases are still unclear. Consequently, in-depth studies on its ability to inhibit inflammation and immune response are needed to consider IL-37 in the treatment of certain diseases. Therefore, this review summarized the broad anti-inflammatory properties of IL-37 in inflammatory diseases, autoimmune diseases, and cancer based on the research progress in recent 5 years.

### IL-37 Transcripts and IL-37 Isoforms

The gene encoding IL-37 is located on the chromosome 2q12-13 that is very close to the regulatory regions of IL-1a and IL-1β genes (5). This specific location may be crucial for the role of IL-37 as an inflammatory response inhibitor (5). The specific activity and relative abundance of each of the five transcripts (IL-37a-e) of the human IL-37 gene are still unclear (4). IL-37b includes 5 of the 6 exons of the IL-37 gene except for exon 3 and it is the most complete, abundant, and studied among all its isoforms (6, 7). IL-37 isoforms a, b, and d share exons 4, 5, and 6, and encode functional proteins involved in the formation of the beta-fold barrel structure essential for the extracellular functional activity of the recombinant IL-37 (4, 8). The IL-37 isoforms c and e lack one or more of these exons, thus, they may encode non-functional proteins (8).

## Regulation of IL-37 Expression and Release

IL-37 is constitutively expressed in several different human tissues and cells, which may help in the maintenance of the immune homeostasis. IL-37 in immune cells is mainly expressed in circulating monocytes, tissue macrophages, dendritic cells (DCs), tonsil B cells, and plasma cells (4, 9). However, the baseline levels of IL-37 transcripts in resting human blood monocytes and DCs are very low due to the presence of an instability sequence in IL-37 mRNA and its short half-life (7). Similarly, the constitutive expression of IL-37 is low or absent in IL-37-tg mice (10). However, IL-37 expression is significantly increased by certain pro-inflammatory stimuli in tissue cells (3). After stimulation, immune cells such as monocytes, DCs, and T cells express IL-37, although the vast majority of peripheral blood mononuclear cells (PBMCs) IL-37+ are monocytes (81%-91%) (9). To sum up, although its constitutive expression is relatively low, the inducible expression of IL-37 can exert a powerful anti-inflammatory effect or immune regulatory role.

The expression of IL-37 in autophagic cells is associated with LC3 conversion (LC3-II/I ratio) (11). In the sequence of the IL-37 promoter, there are the binding motifs of AP-1 and p65 (11). Induced IL-37 expression is associated with an increase in the phosphorylated form of Erk1/2 and AP-1, and could be completely inhibited by Erk1/2 inhibitors or enhanced by Erk1/2 agonists (11). In monkeys, IL-37 expression is increased by chloroquine, and it is negatively associated with CD4 proliferation and phosphorylated STAT3 (11). Hence, upon LPS stimulation, autophagy-modifying reagents (rapamycin and chloroquine) increase the expression of IL-37 through the LC3, Erk1/2 and NF-κB/AP-1 pathways. The pro-inflammatory stimuli increase the production and secretion of IL-37 in the cytoplasm. However, IL-37 precursor does not contain the classical signal peptide at the N-terminus that promotes its translocation into the secretory pathway (4). The presence of extracellular IL-37 precursors, the main form of extracellular IL-37, suggests that this cytokine has a release mechanism that has nothing to do with conventional secretory pathways or cell death (12). It is still unknown whether the IL-37 precursor requires the action of extracellular enzymes to form the “mature” form.

## IL-37-Dependent Molecular Responses on Target Cells

**Figure 2:**
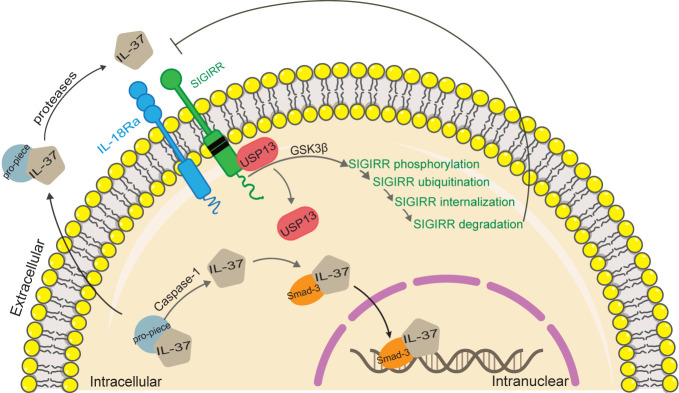
Functional mechanisms of IL-37. IL-37 is a dual function cytokine. As regard the intracellular activity of IL-37, the pro-inflammatory stimuli increase the production of intracellular IL-37 precursor, and trigger the activation of caspase-1, which in turn cleaves IL-37 precursor into mature IL-37. Mature IL-37 binds to phosphorylated Smad-3, forming a complex that translocates into the nucleus, where it regulates gene expression. As regard the extracellular activity of IL-37, both mature and precursor forms of IL-37 are secreted by an unknown mechanism. Extracellular proteases process IL-37 precursor outside the cell, which in turn binds with IL-18Rα and recruits IL-1R8 to form complex exerting the extracellular function of IL-37. IL‐37 induced activation of GSK3β, which plays a role of feedback control of IL‐1R8/Sigirr abundance. Activation of GSK 3β promotes Sigirr phosphorylation, ubiquitination, internalization, and degradation through disrupting Sigirr association with USP13.

IL-37 is a dual-function cytokine since it exerts its anti-inflammatory effects from its extracellular location by binding the surface membrane receptors, and from its intracellular location by the translocation into the nucleus, as shown in **Figure 2**.

### Binding of IL-37 to Receptor Complex

The extracellular IL-37 forms a complex with IL-18 receptor α (IL-18Rα) and IL-1 receptor 8 (IL-1R8) on the cell surface, thereby transducing anti-inflammatory signals. Unlike IL-18, the binding of IL-37 to IL-18Ra does not recruit IL-18Rβ chain to form a functional IL-18 receptor complex (13–16). Inversely, the orphan decoy IL-1 family receptor IL1-R8 is recruited to form the IL-37/IL-18Rα/IL1-R8 complex which decoys MyD88 and limits signaling downstream to IL-1 family and TLR (17). IL-1R8 is necessary for IL-37 to exert its anti-inflammatory effects (1, 18–20). However, IL-1R8/Sigirr is not stable in response to IL-37 treatment (21). IL-37 induces Sigirr degradation in the ubiquitin-proteasome system through site-specific ubiquitination, which can be reversed by a deubiquitinase, USP13 (21). In a recent study, IL-37 induced activation of glycogen synthesis kinase 3β (GSK3β), which plays a role of feedback control of IL-1R8/Sigirr abundance (22). Activation of GSK3β promotes Sigirr phosphorylation, ubiquitination, internalization, and degradation through disrupting Sigirr association with USP13 in lung epithelial cells (22).

In addition, IL-18 binding protein (IL-18BP) can remove soluble IL-18 from the extracellular space thus preventing its binding to the receptor, consequently exerting an anti-inflammatory effect. However, the anti-inflammatory effects of IL-18BP are lost when its level increases probably because IL-18BP binds to IL-37 consequently inhibiting the anti-inflammatory effects of IL-37 itself (15, 23). In conclusion, IL-37 anti-inflammatory effect depends on IL-18R, IL-1R8, and IL-18BP.

Certainly, we need to be aware that IL-37 exists in two forms: a monomer of 22 kDa and a dimer of 42 kDa (3). The biological functions of IL-37 depends on the formation of extracellular IL-37 dimers. Very low concentrations *in vivo* (1 μg per mouse) of recombinant IL-37 are enough to exert its effective and ideal anti-inflammatory effects (24, 25). However, high concentrations of IL-37 had a weak inhibitory effect on the expression of inflammatory cytokines (26). This low-dosing effectiveness of IL-37 may be related to the spontaneous formation of homodimers of IL-37. Dimers may limit the biological activity of IL-37b by reducing the steric affinity for IL-18Rα or by blocking the recruitment of the IL-1R8 co-receptor (27), which may be considered as an auto-regulatory mechanism that limits an excessive immunosuppression. Interestingly, a single amino acid mutation in the IL-37 dimer interface leads to the formation of stable IL-37 monomers, which are maintained at high micromolar concentrations (26). Besides, the anti-inflammatory activity of these IL-37 monomers in many cell types is higher than that of native IL-37 (26). Additionally, IL-37 is a heparin binding protein, and heparin secreted by mast cells blocks the biological activity of IL-37 by promoting its homodimerization (26, 28), suggesting that molecules selectively inhibiting the secretion of mast cell mediators can be used as new therapeutic agents together with IL-37.

### IL-37 Receptor-Independent Mechanisms

Since IL-37 does not contain the nuclear localization sequence, it needs other factors to translocate into the nucleus, thereby regulating the expression of the target gene. IL-37b, c, d, and e have a caspase-1 cleavage site at the aspartic acid (D20) of exon 1, and the caspase-1 cleavage is necessary for IL-37 nuclear translocation (12, 29). After cleaved by caspase-1, the carboxyl domain of IL-37 combines with Smad3 to form a complex. The phosphorylation of Smad3 enables the translocation of IL-37 into the nucleus, where it inhibits the expression of inflammatory genes (30, 31).

The inhibitory effect of IL-37 on LPS-induced MAP kinase and NF-κB activation was reduced or lost in macrophages from IL-37-tg mice carrying the mutation of aspartic acid (D) to alanine (A) at the amino acid 20 (IL-37D20ATg) (32). However, the loss of nuclear translocation does not prevent IL-37 from exerting its anti-inflammatory effect, because IL-37D20A protein is still able to bind to its receptor, consequently exerting the inhibition of innate inflammation (32). Therefore, IL-37 can exert its anti-inflammatory effects through extracellular or intracellular mechanism, although it is still not clear which conditions and/or factors are determining the use of one mechanism instead of the other. Studies aiming at elucidating the above aspect on IL37-tg mice and humans are and will be essential to develop effective approaches in the clinical use of IL-37.

In a recent study, the protective action of IL-37 on the damage after spinal cord injury (SCI) is lost when the extracellular receptor IL-1R8 is missing, while locomotor skills and myelin sparing after SCI are significantly improved in IL-37D20ATg (33). The recombinant IL-37 protein in the presence of IL-1R8 is effective when administered in the lesion site but not systemically (33), suggesting that the nuclear translocation is not required for the beneficial effect of IL-37 on SCI damage, while the extracellular signaling of IL-37 is essential to exert a neuroprotective effect.

### IL-37-Dependent Intracellular Signaling

**Figure 3:**
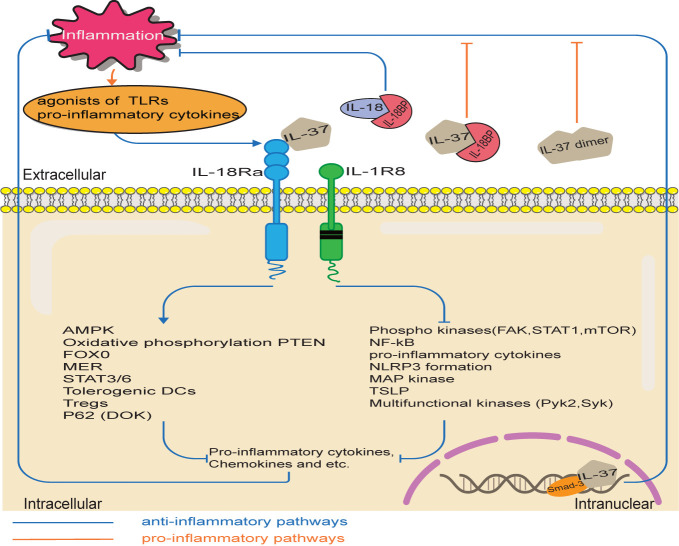
IL-37 signaling pathways. The pro-inflammatory stimuli upregulate IL-37 expression, which in turn inhibits inflammation through various potential pathways. Intracellularly, IL-37/Smad3 complex reduces the inflammatory pathways and increases the production of anti-inflammatory cytokine. Extracellular IL-37 binds to IL-18Rα/IL-1R8. Therefore, pro-inflammatory pathways are inhibited, while anti-inflammatory pathways are activated. However, the bind of an excessive amount of IL-18BP with IL-37 reduces the anti-inflammatory activity of IL-37 and IL-18BP. Moreover, high concentrations of IL-37 had a weak inhibitory effect on the expression of inflammatory cytokines, because of the spontaneous formation of homodimers of IL-37.

During inflammation, IL-37 regulates the activation of various signaling phosphokinases, thereby exerting anti-inflammatory effects (3), as shown in **Figure 3**. IL-37 significantly reduces the activation of pro-inflammatory signaling mediators, including FAK, STAT1, mTOR, p53, p38, paxillin, Pyk2, Syk, SHP-2, and AKT (4). Moreover, anti-inflammatory mediators including the phosphatase PTEN are up-regulated in IL37-tg cells, thus inhibiting the inflammation mediated by the PK3 kinase, mTOR, MAPK and FADK pathways (34).

### IL-37 Genetic Variants

Gout is a severe joint inflammation mediated by IL-1 and induced by the local accumulation of monosodium urate crystals. L37 common variants are related neither with gout nor with circulating IL-1β levels or IL-1β production by PBMCs stimulated with MSU/C16.0 (35). However, four rare IL-37 variants were found in six gout patients: p.(A144P), p.(G174Dfs*16), p.(C181*) and p.(N182S), but none of them was found in healthy controls (HCs) (35). These IL37 rare variants clustered in the functional domain of IL-37 in the exon 5, can lead to abnormal protein structure and function (35). The p.(N182S) variant is associated with genetic susceptibility to gout in hyperuricemic individuals of Polynesian ancestry (35). The p.(C181*) variant led to a loss of anti-inflammatory function and increased cytokine production *ex vivo* (35). However, the treatment with recombinant IL-37 results in the inhibition of MSU crystal-induced joint inflammation in wild type mice (35). Thus, IL-37 appears to be a relevant mediator in the pathogenesis of gout, and the recombinant form can be considered as a potential therapeutic agent to combat gouty arthritis.

## Pathophysiological Effects of IL-37 on Different Cell Types

### *In Vitro* and *In Vivo* Anti-Inflammatory Effects of IL-37

Macrophage-expressed IL-37b reduces pro-inflammatory gene and protein expression upon various inflammatory stimuli relevant to atherosclerosis (36). However, knockdown of IL-37 significantly increased the LPS-induced inflammatory gene and protein expressions in WISH cells, which were reversed by administering recombinant human IL-37 (rhIL-37) (37). Similarly, administration of IL-37 neutralizing antibodies increases the production of inflammatory factors in LPS-Stimulated PBMCs (25). These results suggest one more time the biological function of endogenous IL-37 in the inhibition of inflammatory cytokine production.

IL-37 suppresses the production of IL-1β mediated by NLRP3 and AIM2 inflammasome and that of IL-18 mediated by the NLRP3 inflammasome (38). Although IL-37 does not affect the LPS-induced the expression of the mRNA of IL-18 or inflammasome components, macrophages derived from IL-37-transgenic bone marrow inhibit IL-1b mRNA by up to 83% at steady-state and inhibit LPS-induced IL-1b compared with their wild-type counterparts (38). In addition, IL-37 inhibits the oligomerization/speck formation (which is a step in inflammasome activation and subsequent caspase-1 activation) and pyrolysis (-50%) of apoptosis-associated speck-like protein containing a CARD induced by nigericin and silica (38). Moreover, IL-37d downregulates the expression of NLRP3 at the priming step through the suppression of NF-κB activation by transcriptional profiling, and inhibits NLRP3 inflammasome activation (39). The use of Si-IL-1R8 and MCC-950, a potent and selective inhibitor of the NLRP3 inflammasome, further demonstrates the vital role of IL-1R8 and NLRP3 in the anti-inflammatory effects of IL-37 (40). Accordingly, IL-37d inhibits NLRP3 inflammasome over-activation by regulating NLRP3 transcription through a signaling pathway mediated by IL-1R8 receptor, exerting its anti-inflammatory effects through the inhibition of inflammasome activity. Thus, IL-37 can be considered as a potential agent in the treatment of inflammasome-dependent diseases.

The anti-inflammatory effect of IL-37 has been widely demonstrated not only *in vitro* and in IL-37 transgenic mice, but also in animal models of specific diseases (18, 20, 25, 41), when treated with IL-37. In a recent study, compared with vehicle treatment, IL-37 (1μg/mouse) treatment of mice improves insulin sensitivity and ameliorates obesity-induced inflammation in adipose tissue after 22 weeks of high fat diet (24). Administration of IL-37 decreases plasma insulin levels and pancreatic islet mass possibly by activating AMPK and inhibiting mTOR. Thus, the anti-inflammatory effects of IL-37 can alleviate established metabolic disturbances during obesity.

Since the IL-37 precursor is processed intracellularly and extracellularly *in vivo*, the N-terminus of the naturally occurring IL-37 is subjected to considerable variation, thus, its functional role is unclear. The administration of IL-37 isoforms with different N-terminal ends *in vivo* and *in vitro* revealed the biological complexity of their function. For example, the administration of recombinant IL-37b with the N-terminal end at valine 46 (46–218) *in vivo* is more effective in suppressing inflammation compared with the original IL-37b precursor (14, 25). The same more effective effect is obtained with the use of recombinant IL-37a with the N-terminus at lysine 27 *in vitro* (25). Therefore, these results highlighted the importance of identifying the amino acid sequence that provides the most effective anti-inflammatory effects to develop the most effective IL-37 therapeutic agent.

### Regulation of Cell Metabolism by IL-37 Balance

Otto Heinrich Warburg was the first to describe the choice of cancer cells to obtain energy from anaerobic glycolysis instead of oxidative phosphorylation to produce more ATP necessary for a more rapid cell growth, which is indeed called the Warburg effect. Subsequently, the Warburg effect was also observed in macrophages after LPS stimulation (42), and was associated to an increased level and phosphorylation of mTOR and a decreased activity of AMPK. It was found that IL-37 can reverse the Warburg effect in target cells by inhibiting mTOR and activating AMPK (3, 34, 43).

Fatigue is a common manifestation of chronic inflammatory diseases. The administration of recombinant IL-37 to mice suffering from fatigue induced by inflammation improves their resistance to exercise thanks to the activation of AMPK, which consequently induces metabolic reprogramming (1). Moreover, IL-37 treatment also markedly improved exercise tolerance in healthy mice, which was not secondary to suppression of the inflammatory response (1). These effects are related to the increased rate of oxidative phosphorylation in the mitochondria of the treated animals (1). Thus, IL-37 may be a potential target in the treatment of inflammation-induced fatigue.

Aging is related to vascular endothelial dysfunction, decreased resistance to exercise, and impaired systemic glucose metabolism. The results on experimental animals revealed that the treatment with recombinant IL-37 enhances vascular endothelial function by increasing the bioavailability of nitric oxide compared with the vehicle-treated mice (44). In addition, the treatment with recombinant IL-37 enhances the resistance to exercise by 2.4 times and the ratio of phosphorylated AMPK to AMPK (which is an indication of AMPK activation) in the quadriceps muscle by 2.9 times (44). Recombinant IL-37 treatment also improves systemic insulin sensitivity and glucose tolerance, and modifies the metabolites related to NO synthesis and fatty acid metabolism (44). Thus, IL-37 can be used as a potential agent to improve various physiological functions in the elderly people.

Cell metabolism also depends on the regulation of IL-1R8 by exogenous IL-37 (34), as suggested by the observation that the inhibition of mTOR is mediated by IL-1R8 in Th17-polarized T cells (45). In addition, the effects of IL-37 on exercise tolerance are mediated by IL-1R8 (1). Consequently, IL-37 not only exerts significant anti-inflammatory effects, but also regulates the balance of cell metabolism.

### Effects on Mesenchymal Stem Cells by IL-37

Extracellular IL-37 increases the osteoblast-specific gene expression, the amount of mineral deposits, and the alkaline phosphatase activity of MSCs (46). However, the inhibitors of the PI3K/AKT signaling pathway partially reduced the osteogenic differentiation of MSCs enhanced by IL-37 (46), suggesting the involvement of this pathway in the osteogenic differentiation of MSCs induced by extracellular IL-37.

IL-37 gene-modified MSCs (IL-37-MSCs) can distinctly inhibit intestinal ischemia reperfusion injury (IRI) by migrating to the damaged tissue (47). Indeed, the treatment with IL-37-MSCs on IRI rats strengthens gut barrier function and reduced the local and systemic level of the inflammatory cytokine IL-1β, as compared with rats treated with MSCs or recombinant IL-37 (47). In addition, IL-37-MSCs treatment in IRI rats significantly decreases tissue damage due to NLRP3, the downstream targets such as cleaved caspase-1, IL-1β, and IL-18, and IL-1β- and IL-18-related pro-inflammatory mediators IL-6 and TNF-α mRNA expression, suggesting that NLRP3-related signaling pathway could be associated to the protection mediated by IL-37-MSC (47). Therefore, the modification of the IL-37 gene significantly improves the protective effect of MSCs against intestinal IRI.

### IL-37-Induced Effects on Macrophages

The imbalance of M1 and M2 macrophage polarization can affect the intensity of the inflammatory responses. Calcific aortic valves present a higher amount of M1 macrophages and less IL-37 expression compared to normal valves (48). RhIL-37 down-regulates the expression of inducible nitric oxide synthase, CD11c, IL-6, and monocyte chemoattractant protein 1 (MCP-1) in M1 macrophage *in vitro*, and inhibits their polarization through the suppression of the activation of the Notch1 and NF-κB pathways (48). Moreover, it up-regulates the expression of CD206 and IL-10 in M2 (48). Thus, IL-37 can shift macrophage polarization from the pro-inflammatory M1 phenotype to the anti-inflammatory M2 phenotype.

IL-37 treatment in H1N1 infected BALB/c mice increases their survival rate and body weight, and reduces the pulmonary index, lung injury, and pro-inflammatory cytokines in the bronchoalveolar lavage fluid and lung tissue (49). Since the treatment with IL-37 increases the percentage of macrophages and IL-18Rα+ macrophages, the enhancement of the macrophage function may improve the prognosis of these mice infected with H1N1 (49). In addition, IL-37 suppresses MAPK signaling in H1N1 infected RAW264.7 cells (49). Thus, IL-37 ameliorates influenza pneumonia by the reduction of cytokine production, especially by macrophages, in a MAPK-dependent manner.

### IL-37-Induced Effects on Cholesterol Homeostasis

Atherosclerosis consists of the deposition of lipids and other substances in and on the walls of arteries, thereby forming plaques that restrict the normal blood flow. Mature DCs exert a deleterious effect on the development of atherosclerosis. However, IL-37 suppresses the maturation of DCs induced by oxidized low-density lipoprotein, significantly increases IL-1R8 levels, and decreases TLR4 and p65 levels *in vitro* and *in vivo* (50). The treatment with IL-37 of DCs isolated from IL-1R8 and TLR4-deficient mice results in the loss of the inhibitory effect on the maturation of DCs *in vitro* (50). Thence, IL-37 suppresses the maturation of DCs through the IL-1R8-TLR4-NF-κB pathway and protects ApoE^-/-^ mice against atherosclerosis.

IL-37 rs2708961, rs2723187, and rs2708947 polymorphisms under codominant 1 model are related to low risk of hypercholesterolemia (51). Certain polymorphisms in non- hypercholesterolemia individuals are related to the risk of having high LDL-C and glucose levels, high risk of T2DM, and low risk of having abundant visceral abdominal fat, revealing that some IL-37 polymorphisms are associated to cardiometabolic factors in both individuals with and without hypercholesterolemia (51). Furthermore, the rs2708965, rs2708962, rs6717710, rs2708961, and rs2708960 are related to high levels of C-reactive protein(CRP) in individuals with hypercholesterolemia (51). Thus, IL-37 is also able to regulate cholesterol homeostasis.

### Regulation of Eosinophil- and Mast Cell-Mediated Inflammatory Responses by IL-37

IL-37 mRNA and protein expression is substantially upregulated in nasal epithelial cells of patients with chronic rhinosinusitis with nasal polyps (CRSwNP), compared with control subjects who underwent septoplasty for anatomic variations and did not have other sinonasal diseases (52). IL-37b down-regulates the expression of the TLR3 co-receptor Mex3 RNA binding family member B (Mex3B) in human nasal epithelial cells (HNECs) *in vitro* by the inhibition of polyinosinic-polycytidylic acid-induced production of thymic stromal lymphopoietin (TSLP); this effect is also observed *in vivo* in murine nasal epithelial cells (52). However, the inhibitory effect of IL-37b is abolished by the knock down or overexpression of Mex3B in BEAS-2B cells (52). The level of IL-37 is decreased in the nasal secretions of patients with eosinophilic CRSwNP, because type 2 cytokines suppress the secretion of IL-37 from HNECs (52). The level of secreted IL-37 is negatively associated to the level of Mex3B and TSLP and the eosinophil number in patients with eosinophilic CRSwNP (52). Thus, type 2 cytokines can promote Mex3B activation mediated by TLR3 and subsequent TSLP production through the inhibition of IL-37 secretion in nasal epithelial cells, thus promoting eosinophilic inflammation in patients with CRSwNP.

The interaction between human eosinophils and dermal fibroblasts triggers allergic inflammation in atopic dermatitis (AD). IL-37 levels in AD patients were significantly decreased, together with increased population of eosinophils (53). The serum concentration of involucrin, a keratinizing epithelia protein, in AD patients is significantly higher than that of HCs, which is related to the insufficiency of IL-37 (53). IL-37b suppresses the production of pro-inflammatory cytokines and chemokines in AD, increases autophagosome LC3B protein biogenesis and reduces the ubiquitinated protein p62 associated with autophagy through the activation of AMPK and the inhibition of mTOR (54). In CRISPR/Cas9 human IL-37b knock-in mice, IL-37b significantly alleviates the MC903-induced swelling of the ear tissue and itching sensation, reduced the circulating IL-6 and *in situ* inflammation, decreases eosinophil infiltration in the ear lesion, and significantly upregulates Foxp3+ regulatory T cells (Treg) in ear and spleen (54). Furthermore, IL-37b restores the gut microbiota diversity by the enhancement of autophagy mediated by microbiota metabolites though the regulation of the AMPK-mTOR signaling pathway (54). Thence, IL-37b can significantly reduce allergic inflammation mediated by eosinophils, as well as the diversity of intestinal bacteria and their metabolites in AD through the regulation of autophagy, revealing a potential therapeutic strategy against AD.

Allergic contact dermatitis (ACD) is a T cell-mediated skin inflammatory disease mainly causing Erythema, vesiculation and pruritus. Mast cells (MCs) are involved in the pathogenesis of ACD. The treatment with IL-37 in ACD rats significantly reduces the swelling in their ear, the infiltration of inflammatory cell, IgE levels, the production of IL-33 and inflammatory cytokine, and inhibits MC recruitment (55). IL-37 treatment in rat peritoneal mast cells from ACD rats also decreases the production of IL-6, TNF-α, IL-13 and MCP-1 induced by IL-33 (55) and significantly inhibits NF-κB activation and P38 phosphorylation (55). Moreover, specific Smad3 inhibitors suppress the inhibitory effect of IL-37 on allergic inflammation mediated by MC (55), revealing the role of Smad3 in this inhibitory effect of IL-37. Thus, IL-37 with Smad3 regulation protects against inflammation induced by IL-33-regulated MC by suppressing NF-κB and P38 MAPK activation in ACD rats.

## Regulation of the Innate and Acquired Immunity by IL-37

### IL-37 Regulation of Innate Immune Signaling and Trained Immunity

**Figure 4:**
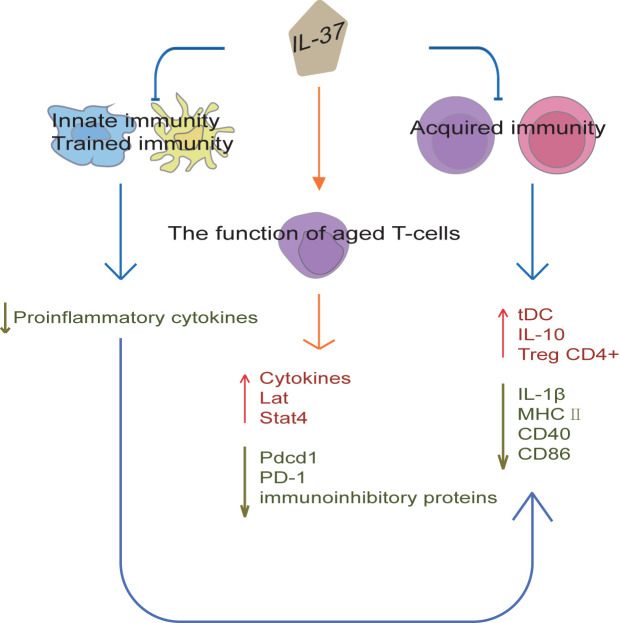
IL-37 regulation of immunity. IL-37 can establish immunological tolerance in the gastrointestinal tract and abolish the protective effects of trained immunity by suppressing pro-inflammatory responses. Moreover, IL-37 can inhibit acquired immunity by producing tolerogenic DCs, which promote Tregs expansion and IL-10 expression, and reduce IL-1β, CD40, CD86 and MHC II expression. The regulation of congenital inflammation by IL-37 also affects acquired immunity. Furthermore, IL-37 can promote cytokine production in aged T-cells and reduce the surface expression of programmed cell death protein 1. IL-37 can also restore a youthful gene expression levels of Pdcd1, Lat, and Stat4 in aged T-cells, and reduce the surface expression of immunoinhibitory proteins. Thus, IL-37 not only regulates the innate and acquired immunity, but also improves aging-associated immunosenescence.

Cytokines are a new immunotherapeutic method that can be potentially used in the diagnosis and treatment of various diseases. IL-37 plays a key role in innate immunity, as shown in **Figure 4**. For example, Intestinal epithelial cells (IEC) are in constant and direct contact with the gut microbiota, and play a central role in coordinating mucosal immunity. IEC are hypo-responsive to bacterial products, which is in part due to their strong expression of SIGIRR. In a recent study, IL-37 (100 pg/ml) attenuated FliC-induced inflammatory responses in human colonoids through inhibition of p38 and NF-κB signaling pathways (56). Moreover, SIGIRR mediates the inhibitory effect of IL-37 in murine colonoids (56). Therefore, IL-37 can promote IEC hypo-responsiveness by suppressing inflammatory signaling, thus regulating innate immune signaling in human and mouse colonic organoids. Zhang et al. reported that a homozygous loss-of-function IL37 variant leading to infantile inflammatory bowel disease (57), which indicates that IL-37 establishes immunological tolerance in the gastrointestinal tract. In addition, IL-37 interference with the congenital protective host response against Candida albicans. The release of TNFα induced by Candida albicans pseudohyphae is significantly decreased in macrophages from IL-37tg mice compared with the release in macrophages from wild-type mice (58). In addition, the recruitment of neutrophils to the site of infection is suppressed in IL-37tg mice, which is associated with an increased mortality, susceptibility to disseminated candidiasis and fungal growth in the kidneys in IL-37tg mice (58). In conclusion, overexpression of IL-37 is not conducive to the early host defense against C. albicans in a murine model of disseminated candidiasis, so the timing of IL-37 expression in the inflamed tissue sites is vital for controlling inflammation in the host.

Trained immunity (TI) is an innate immune memory program induced in monocytes or macrophages by exposure to pathogens, microbial components, or vaccines, as a mechanism to prevent repeated infections (59–61). TI is characterized by immunometabolic changes and histone post-translational modifications, sustaining enhanced production of pro-inflammatory cytokines (62–64). However, excessive activation of the TI programs can cause detrimental inflammation and promote the development of inflammatory diseases (59, 65, 66). On the one hand, administration of IL-37 *in vivo* reduces host pro-inflammatory responses and survival to disseminated candidiasis, thereby abolishing the protective effects of TI (67). On the other hand, IL-37 reverses the immunometabolic changes and histone post-translational modifications in monocytes, thereby inhibiting cytokine production after infection (67). Therefore, IL-37 functions as an inhibitor of TI and can be a potential therapeutic target in immune-mediated pathologies and host responses against pathogens.

### IL-37 Improvement of T-Cell-Mediated Immunity in Aged Backgrounds

Aging-associated declines in immunity pose a risk for the growing aging population, while IL-37 expression levels in human monocytes significantly decreased with age (68). In a recent study, IL-37tg mice mitigates or prevents aging-associated chronic inflammation, splenomegaly, and accumulation of macrophages and DCs in the bone marrow and spleen (68). Moreover, IL-37 promotes cytokine production in aged T-cells and reduces the surface expression of programmed cell death protein 1 (68). IL-37 restores a youthful gene expression levels of Pdcd1, Lat, and Stat4 in aged CD4+T-cells and Lat in aged CD8+ T-cells, and reduces the surface expression of immunoinhibitory proteins (68). Recombinant IL-37 treatment not only improves T-cell function in aged mice, but also improves the efficacy of aged chimeric antigen receptor T-cells which significantly extended the survival of mice transplanted with leukemia cells (68). Thus, IL-37 can boost the function of aged T-cells and overcome aging-associated immunosenescence.

### IL-37 Regulation of DC Immune Function

The number of circulating Tregs in acute coronary syndrome (ACS) patients is reduced, while the population of Th1 and Th17 is increased (69). IL-37-treated DCs obtain tolerogenic DCs (tDCs), such tDCs promote Tregs expansion and decrease the population of Th1 and Th17 when co-cultured with CD4+ T cells (69). Furthermore, IL-37-treated DCs from ACS patients are similar in phenotype and function to IL-37-treated DCs from normal coronary artery patients, and the tolerability of IL-37-treated DCs is very stable (69). Hence, autologous IL-37-treated tDCs may be considered as a potential therapeutic strategy against ACS. Moreover, studies on a mouse skin sensitization model revealed that the adoptive transfer of sensitized DCs from IL37-tg mice to sensitized WT mice results in a significant decrease in immune response compared with that in mice receiving hapten-sensitized DCs from wild-type mice (70). The DCs from IL37-tg mice significantly reduce the expression of IL-1β, IL-6, IL-12, and surface CD40 and MHC class II molecules induced by LPS *in vitro*, and reduce the ability to stimulate naïve T cells and activate antigen-specific T cells (70). Thus, these results suggest that IL-37 inhibits antigen-specific acquired immunity by producing tDCs, with a direct impact in the function of the acquired immune response. Furthermore, DCs isolated from the spleen of IL37-tg mice without antigen sensitization showed a sharp decrease in CD86 and MHC-II expression on the cell surface (3). Thus, the regulation of congenital inflammation by IL-37 also affects acquired immunity.

IL-10 is an important immune factor that inhibits inflammation and immune response through several mechanisms (71). A study revealed that the protective effects of IL-37 is associated to the increased expression of IL-10 in mice with acute pneumonia (70). However, other studies reported that the protective effect of IL-37 have nothing to do with IL-10 (19). Although the expression of IL-10 is increased in DCs from IL37-tg mice subjected to skin sensitization, IL-37 is still able to exert its protective effects in IL-10-deficient mice (18). Thus, these inconsistent results on the potential relationship between IL-37 and IL-10 in the co-regulation of human immune responses suggest that the regulatory mechanisms still need to be clarified.

### Role of IL-37 in Autoimmune Diseases

#### Systemic Lupus Erythematosus

MSCs have immunoregulatory plasticity, and may represent a promising strategy for SLE therapy. The overexpression of IL-37 in MSCs did not change their characteristics of stem cells. These cells increase immunosuppression by inhibiting splenocyte proliferation, decreasing pro-inflammatory factors (IL-1, TNF-, IL-17, and IL-6), and inhibiting autoantibodies (anti-dsDNA and anti-ANA) (72). MSCs overexpressing IL-37 injected into the tail vein of MRL/lpr mice resulted in a better mice survival rate, less SLE signs, significantly decreased pro-inflammatory factors, less total antibody and autoantibody levels, as well as T cell number in serum and kidneys compared with mice that received only control MSCs or IL-37 treatment (72). Expression of IL-37 by MSCs can maintain high levels of serum IL-37 in the mice, and the survival time of MSCs after transplantation is prolonged probably because of the inhibitory effect of IL-37 on the inflammatory microenvironment (72). Thus, the potentiated therapeutic effect of MSCs and IL-37 is probably due to the mutually reinforcing action between them. Genetic modification to overexpress IL-37 may enhance the therapeutic effects of MSCs for SLE.

#### Primary Sjögren’s syndrome

Patients with pSS have a higher serum IL-37 level than those in the HCs, especially pSS patients with positive anti-Ro/SSA and/or anti-La/SSB antibodies (73). The level of total IL-18, free IL-18, and IL-18BP in the serum is higher in pSS patients than in the HCs (73). Importantly, the level of IL-37 in pSS patients is significantly and positively related to the level of antibodies including rheumatoid factor, anti-Ro/SSA and anti-La/SSB, and to the levels of total IL-18 and IL-18BP in the serum (73). Thus, IL-37 may be able to regulate the pathogenesis of pSS.

#### Rheumatoid Arthritis

IL-37 treatment represses the proliferation and migration and induces the apoptosis of rheumatoid arthritis fibroblast-like synoviocytes (RAFLS) (74). Indeed, the expression of apoptosis-related proteins such as BAX and c-caspase-3 are increased, while that of Bcl2 and p-STAT3 are decreased in these cells (74). This increase and decrease of these proteins is regulated by STAT3 (74). Thus, IL-37 inhibits the proliferation and migration of RAFLS, and induces their apoptosis by suppressing the STAT3 pathway. Moreover, in wild-type mice subjected to Streptococcal cell wall-induced arthritis, administration of IL-37(1µg/mouse) suppressed joint inflammation, which were associated with a lower recruitment of neutrophils into the joint (19). Patients with rheumatoid arthritis exhibited a markedly increased synovial expression of IL-1R8, which is required for the anti-inflammatory effects of IL-37 (19).

#### Immune Thrombocytopenia

ITP is an autoimmune disease characterized by low platelet count and heterogeneous bleeding, although severe bleeding in ITP is not completely correlated with low platelet count. One of the major mechanisms triggering ITP is the destruction of platelets mediated by the Fcγ receptor (FcγR). The expression of IL-37 is increased in the plasma of ITP patients, which is correlated with platelet count and the severity of bleeding in ITP (75). Moreover, IL-37 exerts its anti-inflammatory effects on monocytes/macrophages in ITP patients by the downregulation of the phosphorylation in the MAPK, AKT and NF-κB signaling pathway. IL-37 also restores the balance between activating and inhibitory FcγRs, and it reduces the antibody-mediated platelet phagocytosis by monocytes/macrophages (75). Therefore, IL-37 may be a potential biomarker to evaluate disease severity, and provide a new feasible approach in the treatment of ITP.

#### Myasthenia Gravis

Patients with MG possess a much lower IL-37 level in the serum and PBMCs than HCs, which is associated with severer disease (quantitative MG score), and higher follicular Th (Tfh)/Tfh17 and B cell number (76). Tfh and B cells in MG patients have high expression of the IL-37-receptor SIGIRR. IL-37 in MG patients is mainly synthesized by CD4^+^ T cells without overlapping with Th1, Th17, and Tfh subsets (76). In addition, regulatory IL-37^+^ T cells rarely express Foxp3 and CD25, while IL-4 is highly expressed (76). Furthermore, IL-37 directly bound to SIGIRR, inhibits the proliferation and cytokine production in Tfh and B cells, and the autoantibody secretion through the suppression of STAT3 signaling (76). Thus, IL-37 suppresses the autoimmunity in MG *via* direct target of follicular Th and B cells.

#### Hashimoto’s Thyroiditis

Most follicular epithelial cells in tissues from HT patients express IL-37 and SIGIRR, while they are hardly expressed in infiltrating lymphocytes and other inflammatory cells (77). Moreover, IL-37 mRNA expression is significantly higher in PBMC of HT patients than in HCs (77). IL-37 pre-treatment dramatically decreases IL-1β, TNF-α, and MCP-1 mRNA expression in the IFN-γ-stimulated rat thyroid cell line FRTL-5, and remarkably up-regulates IL-4 mRNA expression (77). Thus, IL-37 may have the potential to ameliorate the excessive autoimmune responses in this chronic lymphocytic thyroiditis.

#### Multiple Sclerosis

MS is the most common demyelinating disease of the central nervous system. Transgenic expression of IL-37 reduced inflammation and protected against neurological deficits and myelin loss in experimental autoimmune encephalomyelitis (EAE) mice by acting *via* IL1-R5/IL1-R8 (78). Similarly, administration of rhIL-37 exerted therapeutic actions in EAE mice (78). Although the IL-1R5/IL-1R8 receptor complex is expressed in the PBMC and brains of MS individuals, IL-37 transcripts are relatively insufficient (78). IL-37 may therefore be a potential therapeutic avenue for MS.

## Suppression of Cancer by IL-37

### IL-37-Induced Effects on Tumor Angiogenesis, Migration, and Progression

The protective effect of IL-37 against various cancer types depends on tumor type and stage and IL-37 isoforms. IL-37 treatment increases human umbilical vein endothelial cells (HUVEC) migration and tubule formation, suggesting that IL-37 is a pro-angiogenic factor (79). However, the opposite effect is observed when HUVECs are treated with the supernatant from tumor cell lines overexpressing IL-37, since they undergo apoptosis and their migration and tubule formation is suppressed (79). As regard the *in vivo* effect, IL-37 inhibits tumor angiogenesis in the murine orthotopic hepatocellular carcinoma model, suggesting that it can induce an antiangiogenic effect (79). IL-37 reduces the expression of pro-angiogenic factors and increases the one of antiangiogenic factors in tumor cells (79), as well as decreases matrix metalloproteinase (MMP) 2 expression in SK-Hep-1 and SMMC-7721 cell lines overexpressing IL-37 and murine tumor models (79). The expression of MMP9 and vascular endothelial growth factor (VEGF) is also decreased in SK-Hep-1-venus and SK-Hep-1-IL-37 cells overexpressing IL-37 and murine tumors overexpressing IL-37 under hypoxic conditions (79). Moreover, tumor-associated macrophages (TAMs) promote tumor progression. PBMCs from hepatocellular carcinoma (HCC) patients show M2 polarization and a decreased IL-37 expression (80). IL-37 overexpression inhibits HCC cell proliferation, migration, and invasion by the suppression of M2 polarization through the suppression of the IL-6/STAT3 pathway (80). Furthermore, IL-37 level in HCC samples is positively associated with the infiltration degree of CD1a+ DCs (81). Indeed, IL-37 overexpression in HCC cells is related with the recruitment of more DCs into the tumor tissues by secreting high levels of specific chemokine, such as CCL3 and CCL20, significantly reducing tumor growth (81). Moreover, DCs treated with IL-37 are stimulated to secrete IL-2, IL-12, IL-12p70, IFN-α and IFN-γ, which indirectly enhance the anti-tumor effect of T lymphocytes (81). Thus, IL-37 may contribute to the development of additional therapeutic strategies against HCC.

IL-37b also significantly inhibits MMP and VEGF-A mRNA and protein expression in an endometriosis murine model and in *in vitro* uterine segments (82). In addition, a mature form of IL-37b (IL-37bΔ1-45) effectively suppressed the migration and invasion of endometrial cancer cells by targeting the Rac1/NF-κB/MMP2 signal pathway (83). Furthermore, runt related transcription factor 2 (RUNX2) is a member of the RUNX family that activates genes associated with tumorigenesis and metastasis, promoting tumor cell invasion in cancer. IL-37 overexpression significantly inhibits RUNX2 mRNA and protein expression (84), thus markedly inhibiting cell invasion.

**Figure 5:**
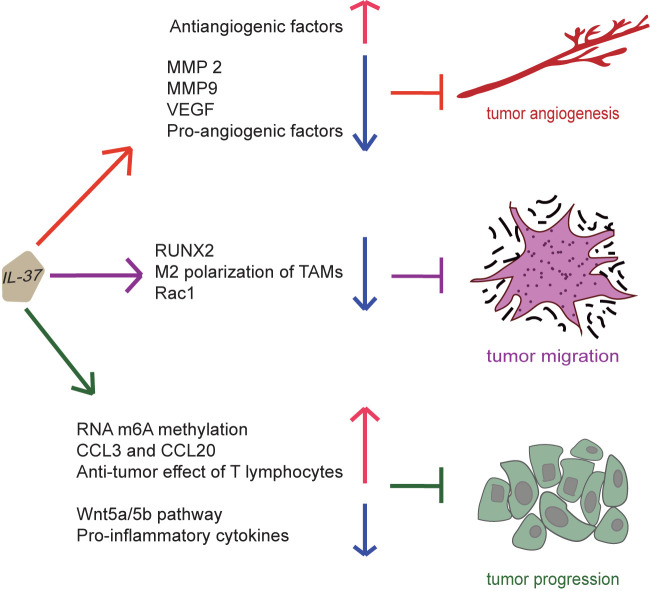
Suppression of cancer by IL-37. IL-37 can induce an antiangiogenic effect on tumor by increasing the expression of antiangiogenic factors and reducing that of pro-angiogenic factors, MMP 2, MMP9 and vascular endothelial growth factor. Moreover, IL-37 can inhibit migration and invasion of the tumor by suppressing M2 polarization of tumor-associated macrophages, activation of Rac1, and expression of runt related transcription factor 2. Furthermore, IL-37 can inhibit tumor progression by promoting RNA m6A methylation, secretion of CCL3 and CCL20, and anti-tumor effect of T lymphocytes and inhibiting Wnt5a/5b pathway and pro-inflammatory cytokine production. Thus, IL-37 exerts inhibitory effects on tumor angiogenesis, migration and progression.

Intracellular mature IL-37 (amino acids 46-218) effectively inhibits the migration of multiple tumor cell types through the inhibition of Rac1 activation; thus, IL-37 loss or decreased expression in lung adenocarcinoma tissues results in tumor metastasis (85). Indeed, intracellular mature IL-37 binds to the CAAX motif in the C-terminal hypervariable region of Rac1, which is normally involved in tumor angiogenesis and metastasis (85). Subsequently, this complex inhibits Rac1 membrane translocation and subsequent downstream signaling (85). Therefore, intracellular mature IL-37 may be considered as a potential therapeutic agent against Rac1 activity and consequent tumor progression. N6-methyladenosine (m6A) is a common transcriptomic modification in cancer, which is involved in the regulation of non-small cell lung cancer formation and metastasis. In a recent study, IL-37 inhibits tumor growth by regulating RNA m6A methylation in lung cancer cells, may downregulate the proliferation by inhibiting Wnt5a/5b pathway in lung cancer cells (86). Therefore, IL-37 can exert inhibitory effects on tumor angiogenesis, migration and progression, as shown in **Figure 5**.

### Protective Effect of IL-37 on Oral Squamous Cell Carcinoma

Chronic inflammation plays a key role in the development of OSCC by the increase of inflammatory cells and cytokines. IL-37 inhibits the pro-inflammatory effects of IL-18 by binding to its receptor. Actually, OSCC patients have high levels of IL-18 and low levels of IL-37 in the serum and PBMCs (87). High IL-18 levels are related to more CD19 ^+^ B cells, while serum IL-37 is related to the decreased percentage of CD3 ^+^ CD8 ^+^ T cells (87). Therefore, serum IL-18/IL-37 balance changes the acquired immune response and affects the progression of OSCC by the modulation of the percentage of CD19 ^+^ B cells and CD3 ^+^ CD8 ^+^ T cells. Thus, IL-37 may be considered as a potential drug against OSCC.

### Diagnostic Value and Prognostic Significance of IL-37 in OSCC

The high levels of IL-18 and low levels of IL-37 in the serum and PBMC of OSCC facilitate the development of advanced tumor stage and lymph node metastasis (the odd ratios of IL-18/IL-37 is 4.903 and 12.613, respectively) (87). Non-cancer individuals can be effectively distinguished from the OSCC patients by the ratio of serum IL-18/IL-37 (cut off value: 2.15) (87). The higher the ratio of IL-18/IL-37, the shorter the overall survival and disease-free survival in OSCC patients, despite this ratio is not an independent prognostic factor (87). Thus, the increased ratio of IL-18/IL-37 in the serum could be used as a potential biomarker for OSCC.

### Diagnostic Value in Oral Leukoplakia

IL-37 expression is higher in OLK than in HCs, probably because of the inflammatory response developed in the body (88). However, IL-37 is less expressed in OLK patients without dysplasia than in those with mild/moderate dysplasia (88). IL-37 overexpression in RAW264.7 cells distinctly suppresses pseudopodia, vacuolization and the expression of IL-6, TNF-α, and IL-1β (88). Thus, IL-37 can be considered as a potential biomarker in the detection of oral tumorigenesis at its early stage and risk assessment of the malignant transformation of premalignant lesions

### Melanoma

The blood of melanoma patients contains lymphocytes (T, B, and natural killer cells) with increased IL-37 mRNA expression, with the highest expression in Treg cells (89). Similarly, Treg cells cultured in melanoma-conditioned media also express IL-37 mRNA and protein (89). In addition, the IL-1-mediated secretome from human melanoma cells, particularly TGF-β, induces IL-37 mRNA expression in human Treg cells (89). Thus, the high expression of IL-37 in specific lymphocyte populations can be considered as a biomarker for immunosuppression induced by tumor.

### Acute Myeloid Leukemia

AML is a malignant hematologic neoplasm, characterized by aberrant proliferation and bone marrow infiltration of myeloid progenitor cells. Serum IL-37 expression was significantly downregulated in newly diagnosed AML patients compared with HCs, and restored in patients in complete remission (90). IL-37 expression was significantly associated with nucleophosmin mutation in AML Patients, and was negatively correlated with IL-6 expression (90). Low IL-37 expression predicted poor prognosis in AML (90). Thus, IL-37 is involved in AML through regulating IL-6 expression.

## IL-37 Involvement in Inflammatory Systemic Diseases and Infections

### Necrotizing Enterocolitis

NEC is a serious and currently incurable intestinal disease mainly affecting preterm infants and it is due to poorly characterized inflammatory pathways. Human and murine NEC intestines have a predominance of type 3/TH17 polarization and the expression of intestinal TLRs is dysregulated (91). Moreover, IL-37 and IL-1R8 are decreased in human NEC epithelia, and IL-37 is also reduced in blood monocytes from infants with NEC and/or low birthweight (91). Although exogenous IL-37 is only moderately effective, IL-37tg mice are effectively protected from intestinal damage and mortality thanks to the regulation of immune homeostasis, TLR repertoires and microbial diversity (91). Thus, type 3 cytokines, TLRs and IL-37 may be used as potential targets for additional and novel therapies to combat NEC. IL-37 alternation in diseases and result of IL-37 treatment are shown in **Table 1**.

**Table 1:** IL-37 alternation in diseases and result of IL-37 treatment.

Diseases	IL-37 levels	Action of IL-37	Pathway	References
Necrotizing enterocolitis	↓	Attenuate intestinal damage and mortality. Regulate immune homeostasis, TLR repertoires and microbial diversity.	Unclear	(91)
Autism spectrum disorder	↑	IL-1β, and CXCL8↓	Unclear	(92)
Temporomandibular joint inflammation	↑	M2 marker ↑ Inflammatory cytokines, pro-inflammatory factor, and synovial M1 marker ↓ Inhibit cartilage degeneration and osteoclast production.	Through IL-1R8 by inhibiting p38, ERK, JNK, and NF-κB.	(40, 93)
Behçet’s disease	Mucocutaneous involvement > systemic involvement	Induce more moderate clinical symptoms. Inhibit TSLP-skin synthesis. Restore homeostasis.	Unclear	(94, 95)
Periodontitis	↑	Suppress alveolar bone loss.	Block osteoclast formation.	(96)
Idiopathic pulmonary fibrosis	↓	Attenuate lung fibrosis. Repress oxidative stress-induced primary mouse alveolar epithelial cells death. Inhibit TGF-β1-induced lung fibroblast proliferation.	Enhance autophagy and inhibit TGF-β1 signaling.	(97)
Spontaneous preterm birth	↓	TNF-α, IL-1β, and IL-6↓ Suppress excessive inflammation, ECM remodeling, and apoptosis.	Inhibit the NF-κB and IL-6/STAT3 pathways.	(98)
Type 2 diabetes mellitus	↓	Inhibit the diabetes development and the promoting effects of miR-657 on inflammatory cytokine production. Increase insulin sensitive.	Inhibit the gut microbiota dysbiosis and NF-κB.	(99, 100)
Calcific aortic valve disease	↓	Suppress the levels of bone morphogenetic protein-2 and alkaline phosphatase as well as calcium deposit formation. Suppress MyD88-mediated inflammatory responses.	Inhibit NF-κB and ERK1/2.	(101–103)
Asthma	↓	Alleviate airway inflammation and remodeling. Suppress the gene transcription of allergic inflammation-related PYCARD, S100A9, and CAMP. Restore normal levels of the eosinophil activators CCL11 and IL-5. Inhibit the increase of Th2. Reduce the mucus, eosinophil infiltration, thickened airway wall, and goblet cells.	Inhibit NF-κB, STAT3 and PI3K-Akt. Target TSLP through NF-κB and ERK1/2.	(104–107)
SARS-CoV-2 infection	↑	IL-6 and IL-8↓ IFN-α↑ Promote biochemical homeostasis. Attenuate inflammation and tissue damage.	By blocking IL-1.	(108–110)
HIV	Steady-state IL-37 mRNA↑	Ameliorate inflammation. Repress HIV replication.	Unclear	(111, 112)
Liver inflammation and fibrosis	↑	Intracellular IL-37 predominantly down-regulates liver inflammation and fibrosis.	Unclear	(113)

### Autism Spectrum Disorder

ASD in children is associated with immune dysfunction and inflammation in the brain. Indeed, IL-37, IL-18 and TNF expression is increased in the amygdala and dorsolateral prefrontal cortex of children with ASD compared to their expression in the brain of non-ASD controls (92). In the same brain areas of children with ASD, IL-18R expression is also increased, while the expression of NTR3/sortilin receptor is reduced (92). The gene expression and secretion of IL-1β and CXCL8 after neurotensin stimulation are inhibited in cultured human microglia from normal adult brains pretreated with hrIL-37 (1 to 100 ng/mL) (92). Moreover, neurotensin, IL-1β and TNF increase the expression of IL-37 in cultured human microglia (92). Thus, IL-37 may be used as a potential therapeutic agent to cure ASD.

### Temporomandibular Joint Inflammation

The synovium and the disc of osteoarthritis patients and the articular cartilage of patients with condyle fractures are characterized by a high expression of IL-37 (93). IL-37 expression is remarkably increased in synovial fluid of patients with synovitis compared with its expression in patients with osteoarthritis and disc displacement, and it is associated with the visual analogue scale score (93). Treatment of chondrocytes with IL-37-pretreated M1-conditioned medium inhibits the expression of inflammatory cytokines (40). In addition, IL-37b inhibits the expression of pro-inflammatory factors *in vitro*, and exerts its anti-inflammatory role through IL-1R8 by the inhibition in the activation of p38, ERK, JNK, and NF-κB (93). However, IL-1R8 silencing upregulates these signals and causes inflammation (93). Furthermore, IL-37 inhibits the expression of synovial M1 markers and cartilage degeneration, and promotes the expression of M2 markers *in vivo* (40). Thus, IL-37 protects temporomandibular joint inflammation by inhibiting inflammation and osteoclast production. Therefore, IL-37b may be a novel and promising therapeutic agent in the treatment of this type of inflammation.

### Behçet’s Disease

BD is a chronic relapse-remitting systemic inflammatory disease with unknown etiology. The release of TSLP and IL-33 is increased in BD patients, and they both dominated the microenvironment in cutaneous lesions with a Th2-type inflammation (94). The expression of TSLP in BD with skin lesions is closely associated to the ratio of the transcription factors GATA3/Tbet (94). However, IL-37 exerts an inhibitory effect on TSLP-skin synthesis and restore the homeostasis (94). Although serum IL-37 level in BD patients is not significantly different from its level in HCs, and is not associated with disease activity, the level of IL-37 was higher in mucocutaneous tissue than in the systemic environment (95). Thus, IL-37 may ameliorate the etiopathogenesis of BD by inducing more moderate clinical symptoms.

### Periodontitis

Periodontitis is a common chronic inflammatory oral disease induced by the interaction between pathogen oral microorganisms and the host immune system. CD138^+^ CD38^+^ plasma cells, the main immune cell type in Chronic Periodontitis gingival tissues, produce IL-35 and IL-37 (96), and experiments *in vitro* demonstrated that human recombinant form of these two cytokines exerts a dose-dependent inhibitory effect on osteoclast formation (96). IL-37-producing plasma cells (CD138^+^CD38^+^P_IL-37_) expressing IL-37 and IL-37/IL-35-coproducing plasma cells (CD138^+^CD38^+^P_IL-35/IL-37_) expressing both of them (96) are denoted as IgG^+^ plasma cells, and reduce periodontitis by suppressing the loss of alveolar bone by blocking osteoclast formation (96). Thus, IL-37 and IL-35 may represent a potential therapeutic agent in the treatment of periodontitis.

### Idiopathic Pulmonary Fibrosis

IL-37 protein expression is significantly reduced in alveolar epithelial cells (AECs) and alveolar macrophages in IPF patients compared with its expression in the same types of cells in HCs (97). IL-37 markedly represses mouse primary AEC death induced by oxidative stress in a dose-dependent manner, while knockdown of IL-37 markedly promotes the death of human lung cancer cells derived from AEC (A549 cells) (97). In addition, IL-37 inhibits TGF-β1 signaling and TGF-β1-induced lung fibroblast proliferation, and enhances beclin-1-dependent autophagy in IPF fibroblasts (97). Thus, the progression of IPF may be associated to a low level of IL-37, and its ability in reducing lung fibrosis is exerted by the induction of autophagy of fibroblasts and the regulation of TGF-β1 production.

### Spontaneous Preterm Birth

The inflammatory imbalance at the maternal-fetal interface promotes the over-secretion of inflammatory factors by human amniotic epithelial cells, and induces apoptosis of human amniotic epithelial cells and extracellular matrix (ECM) degradation, leading to preterm birth. Human peripheral plasma and fetal membranes of sPTB show a decreased IL-37 expression (98). In addition, IL-37 inhibits the production of TNF-α, IL-1β, and IL-6 in WISH cells (98). IL-37 silencing significantly increases LPS-induced apoptosis and activity of MMP 2 and 9 in WISH cells (98). Thus, IL-37 suppresses excessive inflammation, ECM remodeling, and apoptosis by the inhibition of TNF-α, IL-1β, and IL-6 in the cells of the fetal membrane (98).

### Type 2 Diabetes Mellitus

IL-37 is highly concentrated in the serum of the insulin therapy sensitive group compared with the insulin therapy resistant group, and this high expression is associated with a less severe gut microbiota dysbiosis (99). Besides, IL-37 overexpression in model mice inhibits the dysbiosis of gut microbiota and the development of diabetes (99). Moreover, microRNAs (miRNAs) are involved in the development of gestational diabetes mellitus (GDM). The expression of miR-657 is increased, while that of IL-37 is decreased in patients with GDM, thus, miR-657 is negatively associated with IL-37 (100). MiR-657 can target IL-37 and regulate it and increase the proliferation of mononuclear macrophages (100). The treatment with exogenous recombinant IL-37 of LPS-induced mononuclear macrophages significantly inhibits the promoting effects of miR-657 on inflammatory cytokine production and the activation of NF-κB (100). On the one hand, high IL-37 levels in the serum increase insulin sensitivity in elderly type 2 DM patients by the inhibition of the gut microbiota dysbiosis. On the other hand, IL-37 can prevent the effect of miR-657 in macrophages.

### Calcific Aortic Valve Disease

Calcific aortic valve disease is a chronic inflammatory process. Aortic valve cusps (AVCu) express lower IL-37 and increased TLRs levels than mitral valve leaflets (MVL) (101), which may explain the higher incidence of calcification of AVCu than MVL. Aortic valve interstitial cells (AVICs) of diseased aortic valves express greater levels of osteogenic factors following the stimulation of TLR 2 or 4, partly due to a relative lack of IL-37 (102). Treatment of diseased AVICs with rhIL-37 suppresses the levels of bone morphogenetic protein-2 and alkaline phosphatase as well as calcium deposit formation by inhibiting NF-κB and ERK1/2 (102). Moreover, mice expressing human IL-37 exhibit significantly less aortic valve thickening when subjected to a TLR4 agonist or high fat diet (102). Furthermore, IL-37 suppresses MyD88-mediated inflammatory responses in AVICs (103). Thus, IL-37 has therapeutic potential for calcific aortic valve disease.

### Asthma

Asthma is a common respiratory inflammatory disorder disease of childhood, characterized by airway inflammation and airway hyper-reactivity. Airway smooth muscle cells (ASMCs) play an important role in this disease. On the one hand, IL-37 alleviates airway inflammation and remodeling in ovalbumin -induced asthma *via* inhibiting the activation of NF-κB and STAT3 signalings (104). On the other hand, IL-37 not only targets TSLP through NF-κB and ERK1/2 signaling pathways (105), but also may act on tracheobronchial epithelial cells to inhibit fibroblasts and AMSC from producing CCL11, thereby alleviating house dust mite(HDM)-induced asthma (106).

Importantly, IL-37b significantly inhibits the production of inflammatory factors in the co-culture of human primary eosinophils and human bronchial epithelial BEAS-2B cells after the stimulation of bacterial TLR-2 ligand peptidoglycan (107). IL-37 also antagonizes the activation of NF-κB, intracellular PI3K-Akt, and ERK1/2, and suppresses the gene transcription of allergic inflammation-related PYCARD, S100A9, and CAMP (107). In humanized NOD/SCID mice with HDM-induced asthma, the intravenous injection of IL-37b restores normal levels of the eosinophil activators CCL11 and IL-5 in the plasma, and inhibits the increase of Th2, cytokines related to asthma such as IL-4, IL-6, and IL-13, and the level of inflammatory IL-17, CCL5, and CCL11 in lung homogenate (107). IL-37b also reduces the mucus, eosinophil infiltration, thickened airway wall, and goblet cells (107). Thus, IL-37 may exert an anti-inflammatory effect on human allergic asthma, mediated by the above signaling cascades in human eosinophils.

### SARS-CoV-2 Infection

SARS-CoV-2 induces acute severe lung inflammation through IL-1, causing cytokine storm in COVID-19. SARS-CoV-2 infection is characterized by an increased level of IL-37 in the plasma (108). Higher early IL-37 response is associated with an earlier viral RNA negative status, chest CT image improvement, and cough relief compared to the patients with lower IL-37 level in the plasma (108). Moreover, higher IL-37 is associated with lower IL-6 and IL-8 and higher IFN-α, and promotes biochemical homeostasis compared to the low-IL-37 group, while low IL-37 combined with high CRP and IL-8 levels predicts a poor clinical prognosis (108). IL-37 administration attenuates lung inflammation and damage of the respiratory tissue while maintaining type I IFN, consequently keeping safe the function of vital organs (108). Thus, IL-37, by blocking IL-1 (109, 110), may be a potential therapeutic agent to combat SARS-CoV-2 infection, and IL-37, IL-8, and CRP may be used as biomarkers to recognize severe clinical cases.

### HIV

HIV-1-infected patients show higher steady-state IL-37 mRNA expression in PBMCs compared to the expression in non-infected controls, and this IL-37 mRNA expression is associated with the total viral HIV-1 reservoir (111). Moreover, IL-37 in the serum is higher in treated HIV-infected patients compared with their concentration in untreated HIV-infected patients, and rhIL-37 represses HIV replication in human PHA blasts (112). Furthermore, immune cells show a decrease in the expression of IL-37 signaling co-receptor SIGIRR and its soluble form is increased in the serum in HIV-infected patients, situation that can be reversed in those patients treated with antiretroviral drugs (112). The soluble SIGIRR inhibits the anti-inflammatory effects of IL-37, and soluble SIGIRR and IL-37 concentration is associated with certain clinical parameters of the patients (112). Thus, IL-37/SIGIRR axis is functionally compromised in HIV patients and targeting it may ameliorate inflammation and reduce HIV replication in infected patients.

### Sarcopenia

Sarcopenia, characterized by loss of muscle mass and functions, is a highly prevalent condition associated with inflammation in elderly individuals. Successful rehabilitation for sarcopenia results in a reduction significant of CRP (p=0.04) as well as of IL-18 (p=0.008) and IL-37 (p=0.009) concentration (114). Thus, IL-37 may be used as biomarker to monitor the rehabilitation-associated improvement, and as therapeutic targets in sarcopenia.

### Liver Inflammation and Fibrosis

IL-37tg mice improved survival, and reduced hepatic damage and liver fibrogenesis after bile duct ligation, compared with wild-type mice (113). IL-37tg mice were protected against CCl4-induced liver inflammation, and colitis associated liver inflammation and fibrosis (113). Moreover, transgene IL-37 expression reduces the inflammatory response of murine hepatic stellate cells and Kupffer cells (113). IL-37 overexpression reduced the inflammatory response of IL-1β stimulated human LX-2 stellate cells (113). However, rhIL-37 treatment did not regulate fibrosis pathways after bile duct ligation in mice, LX2 cells or murine hepatic stellate cells (113). Therefore, intracellular IL-37 predominantly down-regulates liver inflammation and fibrosis (113). Furthermore, serum IL-37 levels were positively correlated with disease severity in liver cirrhosis (113), suggesting its potential as a target regulating the course of liver fibrosis.

## Conclusions

IL-37 is a dual function cytokine with both intracellular and extracellular forms that exerts broad and complex anti-inflammatory and immunomodulatory effects, inhibits the excess of inflammation, and prevent tissue damage mediated by inflammation. These effects are due to the suppression in the maturation of some inflammatory cells, production of cytokines, and activation of transcription factors and signaling kinases. Extracellularly, IL-37 binds to IL-18Rα and IL-1R8, forming a complex that transduces anti-inflammatory signals. Intracellularly, the IL-37-Smad3 complex translocates into the nucleus where it regulates the transcription, cell maturation, and cytokine production. IL-37 also regulates cell metabolism by the inhibition of mTOR and the activation of AMPK. Furthermore, IL-37 regulates innate and acquired immunity, and improves aging-associated immunosenescence. In recent years, a role for IL-37 has been discovered in several different diseases, such as autoimmune diseases, cancer, and inflammatory diseases. The amount of IL-37 expression is different in different diseases. Thus, the potential use of IL-37 as a novel therapeutic target may be beneficial in the modulation of the inflammatory, metabolic and immune response as well as cancer development. Although IL-37tg mice are protected from the development of autoimmune diseases, IL-37 expression in these disorders is mainly high and positively associated to the disease activity. Thus, further investigations are necessary to discover the different mechanisms used by IL-37 in different autoimmune disorders. Notably, the pathophysiology of IL-37 can be understood from certain chronic infections and cancer, because they evade the immune response through host anti-inflammatory and immunosuppressive mechanisms. Furthermore, IL-37 subtypes and genetic variants follow distinct underlying mechanisms, and miRNAs are also involved in the regulation of the inflammatory response *via* IL-37 in gestational diabetes. However, the actual information on IL-37 highlights its potential role as a promising candidate in the treatment of inflammatory diseases, autoimmune diseases, and cancer. Nevertheless, clinical complications should be avoided by a strict regulation in the balance between effector immune responses required to remove pathogens and limited tissue damage caused by excessive inflammation.

## Author Contributions

ZS and XT contributed to the conception and design of the current study. ZS was responsible for drafting the manuscript. XT performed manuscript review. All authors contributed to the article and approved the submitted version. XT: Conceptualization, funding acquisition, investigation, methodology, resources, supervision, validation, visualization, and writing-review and editing.

## Funding

This work was supported by grants from the National Natural Science Foundation of China (81771070).

## Conflict of Interest

The authors declare that the research was conducted in the absence of any commercial or financial relationships that could be construed as a potential conflict of interest.
